# Self-reported efficacy in patient-physician interaction in relation to anxiety, patient activation, and health-related quality of life among stroke survivors

**DOI:** 10.1080/07853890.2022.2159516

**Published:** 2023-02-01

**Authors:** Jordy Mehawej, Khanh-Van T. Tran, Andreas Filippaios, Tenes Paul, Hawa O. Abu, Eric Ding, Ajay Mishra, Qiying Dai, Essa Hariri, Sakeina Howard Wilson, Jean-Claude Asaker, Joanne Mathew, Syed Naeem, Edith Mensah Otabil, Apurv Soni, David D. McManus

**Affiliations:** a Department of Medicine, UMass Chan Medical School, Worcester, MA, USA; b Department of Medicine, Saint Vincent Hospital, Worcester, MA, USA; c Department of Population and Quantitative Health Sciences, UMass Chan Medical School, Worcester, MA, USA; d Department of Medicine, Cleveland Clinic, Cleveland, OH, USA

**Keywords:** Atrial fibrillation, smartwatch, patient-physician interaction, anxiety, patient activation, health-related quality of life

## Abstract

**Background:**

Early detection of AF is critical for stroke prevention. Several commercially available smartwatches are FDA cleared for AF detection. However, little is known about how patient-physician relationships affect patients’ anxiety, activation, and health-related quality of life when prescribed smartwatch for AF detection.

**Methods:**

Data were used from the Pulsewatch study (NCT03761394), which randomized adults (>50 years) with no contraindication to anticoagulation and a CHA_2_DS_2_-VASc risk score ≥2 to receive a smartwatch-smartphone app dyad for AF monitoring vs. conventional monitoring with an ECG patch (Cardea Solo^TM^) and monitored participants for up to 45 days. The Perceived Efficacy in Patient-Physician Interactions survey was used to assess patient confidence in physician interaction at baseline with scores ≥45 indicating high perceived efficacy in patient-provider interactions. Generalized Anxiety Disorder-7 Scale, Consumer Health Activation Index, and Short-Form Health Survey were utilized to examine anxiety, patient activation, and physical and mental health status, at baseline, 14, and 44 days, respectively. We used mixed-effects repeated measures linear regression models to assess changes in psychosocial outcomes among smartwatch users in relation to self-reported efficacy in physician interaction over the study period.

**Results:**

A total of 93 participants (average age 64.1 ± 8.9 years; 43.0% female; 88.2% non-Hispanic white) were included in this analysis. At baseline, fifty-six (60%) participants reported high perceived efficacy in patient-physician interaction. In the fully adjusted models, high perceived efficacy (vs. low) at baseline was associated with greater patient activation and perceived mental health (*β* 12.0, *p*-value <0.001; *β* 3.39, *p*-value <0.05, respectively). High perceived self-efficacy was not associated with anxiety or physical health status (*β* − 0.61, *p*-value 0.46; *β* 0.64, *p*-value 0.77) among study participants.

**Conclusions:**

Higher self-efficacy in patient-physician interaction was associated with higher patient activation and mental health status among stroke survivors using smartwatches. Furthermore, we found no association between anxiety and smartwatch prescription for AF in participants with high self-efficacy in patient-physician interaction. Efforts to improve self-efficacy in patient-physician interaction may improve patient activation and self-rated health and subsequently may lead to better clinical outcomes.

**KEY MESSAGES**
1. Higher self-efficacy in patient-physician interaction was associated with higher patient activation and mental health status among stroke survivors using smartwatches.
2. No association between anxiety and smartwatch prescription for AF in participants with high self-efficacy in patient-physician interaction.
3. Efforts to improve self-efficacy in patient-physician interaction may improve patient activation and self-rated health and subsequently may lead to better clinical outcomes.

## Introduction

Atrial fibrillation (AF), the most common cardiac arrhythmia, affects nearly 34 million people worldwide [1]. AF also constitutes a global health problem and is a common cause of cardioembolic stroke, hospitalization, and mortality [2]. Therefore, the detection and early diagnosis of AF is of great importance, especially among stroke survivors where undiagnosed AF is common.

Recently, several commercial wearables have received FDA clearance for AF detection and have been proposed as a promising non-invasive option for long-term arrhythmia monitoring in at-risk populations [3–5]. Despite remarkable advancements in smartwatch devices, several potential barriers, including low activation and engagement, may reduce long-term adherence among older adults asked to use a smartwatch over a long period. Physicians play a large role in influencing patient behavior and engagement in health activities [6], and thus prescription of a smartwatch by physicians may increase the likelihood of both wearing a device for AF monitoring and dealing with the potential stress induced by alerts. In fact, efficacy in patient-physician interaction among non-smartwatch users has been shown to improve patient reported outcomes including decreasing anxiety and improving quality of life [7]. It is unknown, however, whether smartwatch users’ perceived efficacy in their interaction with their physicians might relate to activation, anxiety, and health-related quality of life. Understanding these associations is crucial for the effective deployment of smartwatches among post-stroke smartwatch users for AF monitoring. Efficacy in patient-physician interaction may translate to better responsiveness to providers’ prescription of smartwatch devices and in turn improve patient related outcomes.

In this analysis of data from a randomized trial of smartwatches for detecting undiagnosed AF in stroke survivors, we examined the relations of baseline self-reported efficacy in patient-physician interaction with anxiety, activation, and health-related quality of life.

## Methods

### Study population

Details of the Pulsewatch study has been previously described [8,9]. In brief, the study is a two-phase randomized controlled trial designed to measure accuracy, usability, and adherence to smartwatch devices. Participants were enrolled in this study from a large tertiary care center, the University of Massachusetts Memorial Healthcare Center. To be included in the Pulsewatch study, participants had to: (1) be aged 50 years or older; (2) have a history of ischemic stroke or transient ischemic attack (TIA); (3) be proficient in spoken and written English; and (4) be willing to use the Pulsewatch system for at least 44 days. Exclusion criteria included: (1) inability to provide informed consent; (2) a documented contraindication to anticoagulation (OAC) therapy; (3) a documented life-threatening arrhythmia that required in-patient monitoring; (4) having an implantable pacemaker; or (5) presence of allergy or hypersensitivity to medical grade hydrocolloid adhesives or hydrogel. Written informed consent was provided by participants. Study protocols were approved by the Institutional Review Board at the University of Massachusetts Chan Medical School (H00016067).

Between September 2019 and May 2021, trained research staff screened the electronic medical records (EMR) for patients with future cardiology or neurology clinic appointments and enrolled eligible patients. Research staff used the EMR to collect and abstract participant socio-demographic, clinical, and psychosocial characteristics. The sociodemographic characteristics included age, sex, race, marital status, level of education, and household income. Clinical factors included physiologic parameters (body mass index (BMI), systolic blood pressure (BP), diastolic BP, and heart rate), past medical history (vascular disease, cardiac arrhythmia, valvular disease, diabetes mellitus, chronic obstructive pulmonary disease, renal disease, major bleeding event, congestive heart failure, essential hypertension, obstructive sleep apnea, myocardial infarction, and hyperlipidemia), stroke history (ischemic stroke, TIA, and residual neurological deficits), and medication use (OAC, calcium channel blockers, anti-arrhythmic medications, beta blockers, and statins). Psychosocial characteristics included cognitive impairment, social isolation, anxiety at baseline, and technology engagement including device ownership and app use (daily, never, and other which includes few days a week, at least once a week, less than once a week, and once a month).

Participants completed questionnaires at baseline and were then randomized for Phase I (14-day period) in a 1:3 ratio to control or intervention groups. Participants in the control group received only the mobile cardiac outpatient telemetry (MCOT) patch monitor (Cardiac Insight^TM^), a gold-standard clinical ECG monitor, while participants in the intervention group received the MCOT patch in addition to a smartwatch/smartphone dyad. This 14-day period was designed to measure usability and accuracy. Upon completion of the 14-day period, participants completed the same questionnaires assessed at baseline in addition to some questions on user experience which were required by the intervention group to complete. Participants were then re-randomized in Phase II in a 1:1 ratio where participants in the intervention group were monitored and offered the use of the smartphone/smartwatch dyad for an additional 30 days. This 30-day period was designed to measure smartwatch adherence. Permissions were obtained to use all validated questionnaires (questionnaires were not translated to other languages).

Trained research staff provided detailed training to all intervention group participants as well as their family members or caretakers who were present during the study visit. Research staff also provided technical support, if required, and distributed training materials with detailed instructions for successful use.

### Self-reported efficacy in patient-physician interaction

The Perceived Efficacy in Patient-Physician Interactions (PEPPI), a 10-item validated questionnaire, was utilized to measure self-efficacy in patient-physician interactions at baseline, with scores ranging between 5 and 50. Participants were divided into 2 groups, high efficacy group and low efficacy group. A PEPPI score ≥45 was classified as high perceived efficacy in patient-provider interactions [10].

### Study outcomes

We used the Generalized Anxiety Disorder (GAD)-7 Scale, a standardized and validated 7-item questionnaire to assess anxiety at baseline, 14 days, and 44 days. A score of 5 or higher was classified as presence of anxiety [11].

The Consumer Health Activation Index (CHAI), a validated 10-item survey, was used to examine patient activation, referring to a patient’s engagement in their own health, at baseline, 14 days, and 44 days. A CHAI score ≥ 95 indicated high level of activation. Both outcomes were assessed at baseline and at the time of completion of each respective intervention [12].

The Short Form Survey (SF-12), a validated 12-item questionnaire, was used to examine health-related quality of life at baseline, 14 days, and 44 days. This standardized questionnaire includes physical and mental health-related questions determining the physical and mental component. Physical and mental health component assesses the impact of physical and mental health (i.e. well-being, psychological distress) on an individual’s daily life, respectively. Scores range between 0 and 100 with higher scores indicating a higher self-rated quality of health status [13].

### Data analysis

In this study, we included only participants who received a smartwatch/smartphone dyad. Sociodemographic, psychosocial, and clinical characteristics were compared between participants who reported high efficacy in their physician interactions (PEPPI ≥45) and those who did not (PEPPI <45). Chi-square tests and *t-*tests were used to examine between group differences for categorical and continuous variables, respectively.

We used mixed-effects repeated measures linear regression models to examine the association between PEPPI, at baseline, and changes in anxiety, patient activation, and self-rated physical and mental health status, over the study period. In the adjusted model, we adjusted for confounding variables (including race, systolic blood pressure, medical history of diabetes mellitus, and cognitive impairments) based on their statistical significance (*p*-value <0.05). All statistical analyses were completed using SAS 9.3.

## Results

A total of 93 participants who were randomized to receive a smartwatch-smartphone app dyad for AF monitoring from the Pulsewatch study (*n* = 120) were included in this analysis. Participants were, on average, 64 years old (± 8.9), 43.0% were women, and 88% non-Hispanic white. Three-fifths (*n* = 56) of participants reported high perceived efficacy in patient-physician interaction. Nearly 70% of participants had a college degree or higher, 69% were married, and approximately 85% owned a smartphone. A total of 6 participants had AF detected (incidence ∼7%).

Non-Hispanic White participants and those who were cognitively impaired were more likely to report lower efficacy in patient-physician interaction. Participants with a higher mean systolic blood pressure and those with a medical history of diabetes mellitus were more likely to report higher efficacy in patient-physician interaction (Table 1).

**Table 1.** Baseline characteristics according to self-reported efficacy in patient-physician interaction.

Characteristics	PEPPI <45 (*n* = 37)	PEPPI ≥45 (*n* = 56)	*p* Value
Sociodemographic			
Age, mean, years (SD)	64.1 (8.9)	65.3 (9.1)	0.52
Female sex (%)	14 (37.8)	26 (46.4)	0.52
Race – non-Hispanic White (%)	36 (97.3)	46 (82.1)	**0.05**
Married/Living as married (%)	23 (62.2)	41 (73.2)	0.36
College degree or higher (%)	25 (67.6)	38 (67.9)	0.98
Annual household income ≥75k $ (%)	16 (44.4)	29 (54.7)	0.39
Physiologic parameters			
BMI, mean, kg/m^2^ (SD)	29.2 (6.0)	33.4 (26.3)	0.26
Systolic BP, mean, mmHg (SD)	127.1 (15.0)	134.6 (17.2)	**0.03**
Diastolic BP, mean, mmHg (SD)	77.1 (8.2)	76.3 (8.7)	0.67
HR, mean, bpm (SD)	73.6 (12.6)	73.3 (15.2)	0.90
Past medical history (%)			
Vascular disease	6 (16.2)	18 (32.1)	0.10
Cardiac arrhythmias	8 (21.6)	5 (8.9)	0.13
Valvular disease	4 (10.8)	6 (10.7)	0.98
Diabetes mellitus	5 (13.5)	19 (33.9)	**0.03**
COPD	5 (13.5)	5 (8.9)	0.51
Renal disease	3 (8.1)	6 (10.7)	1.00
Major bleeding event	3 (8.1)	3 (5.4)	0.68
Congestive heart failure	1 (2.7)	5 (8.9)	0.40
Essential hypertension	27 (73.0)	44 (78.6)	0.62
Obstructive sleep apnea	7 (18.9)	17 (30.4)	0.24
Prior myocardial infarction	5 (13.5)	21 (21.4)	0.42
Hyperlipidemia	29 (78.4)	50 (89.3)	0.24
Stroke history			
Ischemic stroke	26 (70.3)	48 (85.7)	0.11
TIA	14 (37.8)	15 (26.8)	0.36
Residual neurologic deficits	12 (32.4)	17 (30.4)	0.87
Medication use (%)			
Anticoagulants	4 (10.8)	7 (12.5)	1.00
Calcium channel blockers	6 (16.2)	12 (21.4)	0.61
Anti-arrhythmic medications	2 (5.4)	0 (0.0)	0.16
Beta blockers	12 (32.4)	28 (50.0)	0.13
Statins	33 (89.2)	52 (92.9)	0.71
Psychosocial characteristics (%)			
Cognitive impairment	32 (86.5)	34 (60.7)	**<0.01**
Social isolation	33 (89.2)	49 (87.5)	1.00
Anxiety via GAD-7 score			
None (0–4)	22 (61.1)	39 (69.6)	0.32
Mild (5–9)	10 (27.8)	12 (21.4)	
Moderate (10–14)	2 (5.6)	5 (8.9)	
Severe (15+)	2 (5.6)	0 (0.0)	
Anxiety via GAD-7 score (%)	14 (38.9)	17 (30.4)	0.49
Technology engagement			
Device ownership			
Smartphone	33 (89.2)	46 (82.1)	0.39
Smartwatch	13 (35.1)	11 (19.6)	0.15
App use frequency			
Daily	28 (80.0)	30 (60.0)	0.22
Never	0 (0.0)	4 (8.0)	
Other	7 (20.0)	16 (32.0)	

The significance of *p*-value is 0.05.

Participants who reported high perceived efficacy had higher patient activation and self-rated mental health status compared to those with low perceived efficacy (Table 2; *β*-estimate 12.0, *p*-value <0.001; *β*-estimate 3.39, *p*-value <0.05, respectively), but high efficacy was not associated with anxiety or self-rated physical health status, after adjusting for confounding variables (Table 2; *β*-estimate −0.61, *p*-value 0.46; *β* 0.64, *p*-value 0.77, respectively).

**Table 2.** Anxiety, patient activation, and self-rated physical and mental health status in relation to efficacy in patient-physician interaction among smartwatch users.

	GAD score			CHAI score			SF-12 PCS			SF-12 MCS		
PEPPI ≥45 (vs. PEPPI <45)	Unadjusted models											
	Estimate	SE	*p* Value	Estimate	SE	*p* Value	Estimate	SE	*p* Value	Estimate	SE	*p* Value
	−0.98	0.66	0.19	**12.8**	**2.3**	**<0.001**	0.26	2.04	0.90	**4.1**	**1.5**	**<0.01**
	Adjusted models^a^											
	−0.61	0.82	0.46	**12.0**	**2.41**	**<0.001**	0.64	2.20	0.77	**3.39**	**1.63**	**<0.05**

^a^Adjusted Variables: race, systolic BP, medical history of DM, and cognitive impairment.

PEPPI: Perceived Efficacy in Patient-Physician Interactions; CHAI: Consumer Health Activation Index; SF-12: Short Form Survey [PCS & MCS: Physical and Mental Health Component (assesses the impact of physical and mental health on an individual’s daily life)].

The significance of the *p*-value is <0.001.

## Discussion

In this randomized controlled trial of older stroke survivors randomized to receive a smartwatch for AF detection, we observed that 60% of smartwatch users reported high efficacy in patient physician interaction. We also found that high efficacy in patient-physician interaction among smartwatch users was associated with improved patient activation and self-reported mental health over the study period.

Our study showed that smartwatch users who reported high efficacy in patient-physician interaction, compared to those who reported lower, had a significant increase in patient activation over the study period. This finding suggests that effective patient-physician interactions may be an important factor in smartwatch users taking a more active role in their health. In a study involving 8,140 chronically ill patients, a higher efficacy in patient-physician interaction was also associated with greater patient activation [7]. This finding is particularly significant in highlighting the importance of an efficient patient-physician interaction in altering patient behavior and approaches towards their care. We hypothesize that smartwatch users with higher efficacy in patient-physician interaction may respond positively to providers’ prescription of smartwatch and adhere to its use for AF monitoring, and, subsequently, may feel involved and playing a more active role in their care.

In the present study, smartwatch users with high perceived efficacy in patient-physician interactions at baseline were associated with improved mental health but not associated with improved physical health. Studies on patient-physician communication showed that effective communication was reported to improve self-rated physical and mental health [14,15]. Different findings with regards to the association between patient-physician interaction and self-rated physical health between our study and these prior investigations could be explained by differences in sample size, the clinical and sociodemographic characteristics of our study populations, and the instruments used to examine efficacy and physical health. Smartwatch users with high efficacy at baseline may respond differently/possibly better to physicians/researchers who ask them to engage in AF monitoring in a sustainable way. They may, in turn, feel better mentally and possibly physically. Conversely, those with lower self-efficacy may struggle and have adverse effects of smartwatch monitoring that are not presently considered. High self-efficacy could have provided an enabling environment where patients feel empowered and involved in their care hence influencing their mental health outcome positively. These findings highlight the importance of establishing improved patient-physician interaction amongst stroke survivors offered smartwatches for AF detection.

To date, there have been reports of anxiety amongst smartwatch users [16] with several possible underlying etiological factors. Our study showed that smartwatch users with high reported efficacy in patient-physician interaction was not associated with anxiety, over the study period. This finding potentially underscores the importance of high efficacy in patient-physician interaction amongst smartwatch users, which is further supported by prior work demonstrating that good patient-provider communication is inversely associated with anxiety levels [17,18].

### Strengths and limitations

Our study has several strengths and limitations. Data was used from a multi-phased randomized control trial which is unique in examining smartwatch-based cardiac rhythm monitoring and patient reported outcomes in post-stroke older adults. In addition, participants included in this study are post-stroke older men and women who are well characterized with respect to sociodemographic, clinical, and psychosocial characteristics as well as self-reported outcomes. Moreover, we utilized standardized, validated instruments including PEPPI, GAD-7, CHAI, and SF-12 to examine self-reported efficacy in patient-physician interaction, anxiety, patient activation, and health-related quality of life, respectively, increasing the validity and reproducibility of our study findings. However, our study has several limitations. First, the study cohort is highly homogenous with regards to race/ethnicity which may limit the generalizability of our findings to other ethnic groups. Second, we examined the association between PEPPI level at baseline and changes in our outcomes over a short duration (44 days). Finally, we monitored the detection of AF and examined patient reported outcomes in a modest size cohort which may limit the generalizability of our findings.

## Conclusions

In this analysis of data from a randomized trial of stroke survivors prescribed smartwatches for AF monitoring, we observed that higher self-efficacy in patient-physician interaction is associated with greater patient activation and mental health. We found no association between anxiety and smartwatch prescription for AF in participants with high self-efficacy in patient-physician interaction. Efforts to improve efficacy in patient-physician interaction among smartwatch users may enhance patient activation and self-rated health, reduce stress, and promote long-term adherence to smartwatches among older adults naïve to use of these devices. Further studies are needed to evaluate whether clinicians can leverage commercial wearables to promote detection of undiagnosed AF in high-risk populations.

## Data Availability

The data that support the findings of this study are available from the corresponding author, [JM], upon reasonable request.
